# Psychological stress during medical internship is associated with inflammatory signatures linked to mental health

**DOI:** 10.1016/j.bbih.2026.101243

**Published:** 2026-04-18

**Authors:** Jasmin Ewert, Laura Meine, Ella McPherson, Sarah Fehr, Tobias R. Spiller, Benedikt Dechow, Gaia Ferrante, Sebastian Hachenberg, Lidya Aslan, Antonia Lüönd, Bogdan Rivoal Mateescu, Birgit Kleim, Flurin Cathomas

**Affiliations:** a Department of Adult Psychiatry and Psychotherapy, University Hospital of Psychiatry Zurich, University of Zurich, Zurich, Switzerland; b Experimental Psychopathology and Psychotherapy, Department of Psychology, University of Zurich, Zurich, Switzerland; c Brain Research Institute, University of Zurich, Zurich, Switzerland

**Keywords:** Psychosocial stress, Immune dysregulation, Mental health, Matrix metalloproteinase-8 (MMP-8), Inflammation, Medical internship

## Abstract

Psychosocial stress is a major risk factor for mental disorders and affects peripheral physiology, including immune function. However, it remains unclear how stress exposure in otherwise healthy individuals alters circulating immune markers in relation to common mental health symptoms. We investigated this question in otherwise healthy medical students (N = 82) during their first medical internship, a naturalistic stressor. In hypothesis-driven analyses, we tested whether longitudinal changes in three predefined inflammatory markers, matrix metalloproteinase-8 (MMP-8), tumor necrosis factor- (TNF)-α, and interleukin-6 (IL-6), were associated with changes in General Health Questionnaire-28 (GHQ) total and subscale scores. We additionally performed exploratory analyses of a broader circulating immune-protein panel to identify other candidate markers associated with symptom change. GHQ scores and circulating immune markers were assessed before the internship and again after three months. The internship period was associated with increased GHQ scores. Greater increases in circulating MMP-8 were associated with greater worsening of GHQ scores, driven primarily by the Anxiety/Insomnia subscale. In contrast, changes in TNF-α and IL-6 were not significantly associated with GHQ total or subscale scores. Exploratory analyses identified several nominal associations with GHQ total scores, but none survived multiple-testing correction and these findings should be considered hypothesis-generating. These findings suggest that MMP-8 may be a relevant immune correlate of stress-related symptom worsening during a naturalistic stressor. Future studies should clarify temporal directionality and determine whether interventions targeting neuroimmune and inflammatory pathways can reduce stress-related mental health symptoms.

## 1 Introduction

Stress is an inevitable part of life and ranges from minor daily hassles to major life events and potentially traumatic stressors. Numerous studies have demonstrated that chronic stress exposure increases the risk for a range of mental disorders, including major depressive disorder (MDD) and anxiety disorders, as well as physical conditions such as cardiovascular disease, cancer, and autoimmune disorders (Kalisch et al., 2017; Hammen, 2005; Bryant, 2019; Otte et al., 2016). This has been shown not only in the context of traumatic experiences, but also in work-related settings, where long-term stress is associated with an increased prevalence of somatic and mental disorders (Kivimaki, 2002; Theorell et al., 2015; Maslach et al., 2001). Particularly relevant are the high rates of stress-related mental health problems in healthcare personnel (Mata et al., 2015). Despite considerable efforts to increase our understanding of the pathophysiology of stress-related behavioral alterations and psychopathology, their biological underpinnings are still not well understood.

There is a close interplay between the brain and the immune system, which can be significantly influenced by stress (Cathomas et al., 2019, 2022; Wohleb et al., 2016). The physiological response to acute stress and potential threats is essential for adapting to environmental changes. The autonomic nervous system and hypothalamic-pituitary-adrenal (HPA) axis play a central role in coordinating the body's response to perceived threats (Herman et al., 2016). Upon exposure to stressors, the hypothalamus releases corticotropin-releasing hormone, which stimulates the pituitary gland to secrete adrenocorticotropic hormone. This, in turn, prompts the adrenal cortex to produce cortisol. Simultaneously, the sympathetic branch of the autonomic nervous system is activated, leading to a cascade of effects, including the release of epinephrine and the mobilization of immune cells from the bone marrow into circulation, where they secrete inflammatory proteins such as cytokines and chemokines (Smith and Vale, 2006; Ulrich-Lai and Herman, 2009). While these mechanisms are adaptive and beneficial in the short term, sustained activation can have negative effects on multiple organ systems, including the brain (Miller and Raison, 2016).

Moreover, multiple lines of evidence show that individuals with mental disorders, including anxiety disorders and MDD, display increased levels of circulating pro-inflammatory cytokines, chemokines, and other inflammatory proteins (Dowlati et al., 2010; Osimo et al., 2020; Goldsmith et al., 2016). Among these, C-reactive protein (CRP), interleukin (IL)-6, and tumor necrosis factor (TNF)-α represent the most frequently studied and most consistently elevated markers in psychiatric research (Baumeister et al., 2014). For example, individuals with clinically significant anxiety exhibit elevated circulating IL-6 levels, and increased IL-6 and TNF-α concentrations have been reported in patients with generalized anxiety disorder and panic disorder compared with healthy controls (Hou et al., 2016; O'Donovan et al., 2010). Similarly, a subgroup of patients with MDD shows elevated plasma IL-6 and TNF-α levels relative to healthy controls, with meta-analyses confirming these elevations (Liu et al., 2012; Hiles et al., 2012; Köhler et al., 2017). Rather than being limited to alterations in single inflammatory markers such as CRP or circulating cytokines, increasing evidence suggests that psychiatric disorders are associated with broader dysregulation across multiple domains of the immune system. These include components of the innate and adaptive immune response (Wohleb et al., 2016; Stuart and Baune, 2014; Pinzi et al., 2025; Dai et al., 2024). Recent studies suggest that patients with MDD show alterations of proteolytic systems such as matrix metalloproteinases (MMPs), which regulate extracellular matrix remodeling and vascular integrity and may contribute to neurovascular and neuroimmune dysfunction (Cathomas et al., 2024; Dickerson et al., 2026), thereby providing a potential mechanistic link between inflammation and symptoms of mental disorders (Pinzi et al., 2025; Levy et al., 2018). However, research into whether these findings may translate to healthy individuals and those facing stressors on a regular basis (e.g. as part of their work) is sparse. Several studies have used specific laboratory-based tests, such as the Trier Social Stress Test Kirschbaum et al. (1993), in healthy populations and have reported increases in several circulating cytokines, such as IL-1β, IL-6, IL-10, and TNF-α (Steptoe et al., 2007; Von Känel et al., 2006; Marsland et al., 2017). Yet, these laboratory-based stressors are limited, e.g., due to their short exposure duration and they may not accurately mirror real-world conditions. Increases in circulating pro-inflammatory proteins were also found when investigating more naturalistic stressors in education- or work-related settings (La Fratta et al., 2018; Lee et al., 2021, 2022; Kim et al., 2016; Duchaine et al., 2021). However, most of these studies again investigated a relatively short period of stress and immune phenotyping was often based on a limited set of commonly used markers, which may not fully reflect the complexity of the immune system.

Immune responses to stress involve coordinated activity across multiple mediators, including cytokines, chemokines, and proteases, which are statistically and biologically interdependent. Analyses focused on single markers may therefore miss system-level patterns of co-regulation. Network approaches model multivariate association structures among immune markers (and between immune markers and symptoms), enabling characterization of immune organization and providing a complementary perspective to univariate marker-outcome associations. Similar network frameworks have been applied to examine relationships among inflammatory markers and psychiatric symptom domains (Moodley et al., 2023; Zainal and Newman, 2023). Because psychosocial stress is unlikely to affect single immune markers in isolation, but rather to induce coordinated changes across interconnected pathways, a network-based approach may help to capture these system-level alterations. To address these gaps, the present study takes a prospective approach to investigate immune system changes in response to real-world stress exposure and link these changes to the development of stress-related symptoms of depression and anxiety as well as negative affect. Specifically, we use the transition into clinical internships among medical students as a real-life and ecologically valid model of stress. This period is widely recognized as psychologically demanding, with previous studies showing that medical personnel commonly experience stress during their training and exhibit a high prevalence of mental symptoms (Grueschow et al., 2021; Rotenstein et al., 2016).

To assess stress-related changes in mental health, we employed the General Health Questionnaire-28 (GHQ), a widely used screening instrument that captures general mental health symptoms across domains including anxiety, depressive symptoms, social dysfunction, and somatic symptoms (Goldberg and Hillier, 1979). The GHQ is suitable for detecting emerging or subclinical symptom changes in populations exposed to stress (Vallejo et al., 2007). In addition, we assessed perceived stress using the Perceived Stress Scale (PSS), which measures the extent to which individuals appraise their lives as unpredictable, uncontrollable, and overwhelming (Cohen et al., 1983). Based on prior evidence linking psychosocial stress to inflammatory activation, we formulated *a priori* hypotheses that longitudinal changes in MMP-8, TNF-α and IL-6 would be associated with changes in general mental health symptoms, as assessed by the GHQ.

## 2 Methods

Participants. Medical students in their fifth year (total duration of medical school in Switzerland is six years) at the University of Zurich, Switzerland, were recruited for this study as they were about to begin their internship year (the first clinical internship in medical school), providing an ecological and well-established model to examine psychological responses to prolonged real-world stress. Previous prospective studies have demonstrated that medical internships are associated with increases in anxiety and depressive symptoms over time, making them a suitable paradigm for investigating stress-related changes in mental health (Grueschow et al., 2021). Inclusion criteria required participants to be physically and mentally healthy (i.e., absence of a current medical or psychiatric diagnosis), magnetic resonance imaging (MRI)-compatible, and fluent in German. Eligibility was assessed during a structured telephone interview prior to study enrollment. This study is part of the Hochschulmedizin Zurich (HMZ) STRESS project, a longitudinal observational study following medical students during their first clinical internship, a reportedly stressful period. In total, 105 students were initially enrolled (Meine et al., 2025). Complete blood samples were available for 82 participants, and all primary and exploratory analyses were conducted on this subset. Ethics approval for the study was obtained from the Cantonal Ethics Commission of Zurich (KEK, 2022-01169) and informed consent was obtained from all study participants. The overall cohort study was preregistered, whereas the present immune-protein analyses were conducted without formal preregistration (McPherson et al., 2023).

Procedure. Baseline measurements (T0) were conducted within one month before students began their first clinical internship. Three months later, during their practical training, participants were reassessed (T1). At both time points, blood samples were collected in a fasted state. Additionally, students completed the GHQ (28-item version) Goldberg and Hillier (1979), the PSS-14 Cohen et al. (1983), the Mainz Inventory of Microstressors (MIMIS) (Chmitorz et al., 2020), and the Pittsburgh Sleep Quality Index (PSQI) Buysse et al. (1989) at each time point. The GHQ is a self-report measure designed to assess general mental health symptoms across four domains: somatic symptoms, anxiety/insomnia, social dysfunction, and severe depression. The PSS-14 is a 14-item self-report questionnaire assessing the degree to which individuals appraise their lives as stressful during the past month. Items are rated on a 5-point Likert scale ranging from 0 (“never”) to 4 (“very often”). Total scores range from 0 to 56, with higher scores indicating greater perceived stress. PSS scores were primarily used to confirm subjective stress exposure across the internship period and to characterize stress levels within the cohort. The MIMIS assesses exposure to everyday microstressors, whereas the item 4 of the PSQI evaluates sleep duration over the past month; for the present analyses, item 4 (habitual sleep duration) was used.

Plasma preparation and analyses. Blood samples were collected from participants at two time points: before (T0) and during (T1) their practical year. Samples were drawn into ethylenediaminetetraacetic acid-(EDTA) containing tubes and centrifuged at 2000 RCF for 10 min. The resulting plasma was aliquoted and stored at −80 °C until analysis. Protein analysis was performed using the SomaLogic SomaScan assay (Gold et al., 2010), a high-throughput aptamer-based; 100 proteins were quantified in plasma. Based on prior literature linking inflammatory processes to psychosocial stress and affective symptoms, three immune markers, MMP-8, TNF-α, and IL-6, were pre-specified for hypothesis-driven analyses (Wohleb et al., 2016; Cathomas et al., 2024; Hassamal, 2023; Doney et al., 2022). In addition, an exploratory panel of 97 candidate proteins representing major pathways involved in stress-response (Supplementary Table 1) was examined. These proteins were selected based on their reported or hypothesized associations with stress-related mental disorders as reviewed by Hassamal and Feng et al. (Hassamal, 2023; Feng et al., 2025) and analyzed to identify additional stress-related immune alterations.

Statistical Analyses. A *post hoc* power analysis was conducted using G∗Power (version 3.1.9.7) Faul et al. (2007) to estimate the achieved statistical power for the linear regression models. The analysis was specified as an F test for linear regression (fixed model, R^2^ increase). Based on an assumed medium effect size (f^2^ = 0.15), an alpha level of 0.017 (adjusted for multiple testing), a total sample size of N = 82, one tested predictor, and five total predictors in the model, the achieved power (1 − β) was 0.85. The corresponding critical F value was 5.96 with 1 numerator and 76 denominator degrees of freedom. This indicates adequate statistical power to detect effects of medium magnitude.

Hypothesis-driven analyses. We used paired, two-sided t-tests to assess whether the changes in GHQ and the PSS, the MIMIS and the PSQI item 4 between baseline (T0) and follow-up (T1) were statistically significant. To assess changes in general mental health symptoms and immune markers before and three months into the internship, delta scores were calculated for GHQ and all analytes (T1 - T0). Primary hypothesis-driven analyses focused on the three pre-specified immune markers (MMP-8, TNF-α and IL-6). Linear regression models were used to assess associations between changes in each marker and changes in GHQ scores. In addition, linear mixed-effects models were fitted using the repeated measures at T0 and T1 to account for within-subject dependence and to make use of all available longitudinal data. To account for multiple testing across the three pre-specified markers, false discovery rate (FDR) correction was applied to the analyses involving the GHQ total Score. In addition, gender-stratified linear regression analyses were conducted for MMP-8. To further assess the robustness of the association between MMP-8 and GHQ, an additional linear regression model was fitted including PSQI item 4 as a covariate.

Exploratory proteomic analyses. To identify additional immune markers associated with general mental health symptom changes beyond the *a priori* hypotheses, linear regression analyses were performed to examine the predictive power of the 97 protein panel using the same modeling approach. To account for multiple comparisons, FDR correction was applied. Proteins that met a nominal uncorrected p-value of <0.05 but did not survive this correction were considered hypothesis-generating findings for future validation.

For both hypothesis-driven and exploratory analyses, values exceeding ±2 standard deviations from the mean delta were excluded from the respective analysis. Given the known variability and skewness of immune marker distributions, this approach was chosen to reduce the influence of extreme values that may reflect transient physiological states or technical variability rather than stress-related immune changes, as this strategy is commonly used in biomarker research to improve robustness and interpretability of statistical analyses (Boerrigter et al., 2017; Jacomb et al., 2018; Genser et al., 2007). The number of participants included in each analysis after outlier exclusion is reported in Supplementary Table 1. All regression and linear mixed models were adjusted for age, body mass index (BMI), gender, and smoking status. All analyses were conducted in *R* (version 4.4.1, 2024-06-14) (R Core Team, 2024; RStudio Team, 2024).

Network analysis. Network analysis was used to explore the interrelations among immune markers and general mental health symptoms as assessed by the GHQ. We utilized the *bootnet* package in R, specifically employing the *estimateNetwork* function with the mgm (Mixed Graphical Models) default setting (Epskamp and Fried, 2018; Haslbeck and Waldorp, 2020). This approach is suitable for datasets containing a mix of variable types, such as continuous and categorical data. To control for potential confounding influences, we applied partial correlation analysis while adjusting for age, BMI, gender, and smoking status. We avoided shrinking weaker connections by setting the regularization parameter, lambda, to 0 (Genser et al., 2007), thereby preserving all potential associations. To assess the stability of centrality estimates, we conducted a case-dropping bootstrap with 1000 iterations. This approach evaluates how centrality metrics hold up when increasing proportions of participants are randomly removed from the dataset, providing a robustness check for the network structure.

## 3 Results

**Fig. 1:**
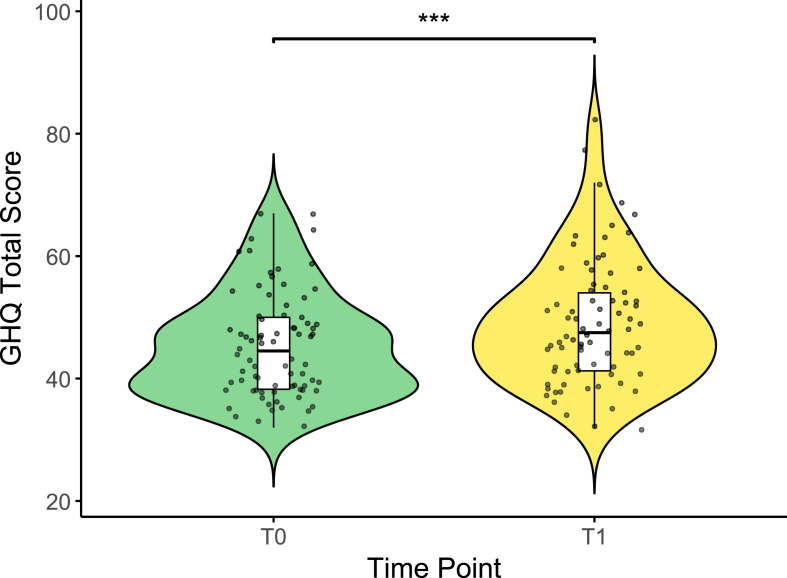
**Increased general mental health symptoms after 3 months of a medical internship.** Distribution of General Health Questionnaire (GHQ) total scores at baseline (T0) and after 3 months (T1). The violin plot illustrates the density of individual data points, with an overlaid boxplot indicating the median, interquartile range, and overall spread of the data. Statistics: Paired two-sided *t*-test (∗∗∗p < 0.001).

Sociodemographic variables of the 82 study participants are summarized in Table 1. We first replicated previous findings (Grueschow et al., 2021) that the real-world stress of a medical internship was indeed perceived as stressful (as shown by increased total scores in the PSS, increased exposure to microstressors as measured by the MIMIS Exposure score, as well as changes in sleep duration assessed by the PSQI item 4, Supplementary Fig. 1) and confirmed that it increases general mental health symptoms as operationalized by an increase in GHQ scores (Fig. 1) after three months compared to baseline.

**Table 1:** **Demographic and health-related characteristics of the study sample (N = 82).** The table summarizes demographic variables including age, body mass index (BMI), gender distribution, and smoking status, as well as psychometric measures assessed at baseline (T0) and follow-up (T1), including the General Health Questionnaire (GHQ) and GHQ subscales, the Perceived Stress Scale (PSS), Mainz Inventory of Microstressors (MIMIS) Exposure, and Pittsburgh Sleep Quality Index (PSQI)-4. Values are presented as mean ± standard deviation (SD). Cohen's d for paired comparisons (T1–T0) is reported as a measure of effect size.

Characteristics	Count/Percentage (N = 82)			
**Demographics**				
Age (years) [Mean ± SD]	23.6 ± 1.2			
Gender				
Female [%]	72			
Male [%]	28			
**Anthropometrics and Lifestyle**				
BMI [Mean ± SD]	22.3 ± 3.0			
Smokers [%]	3.7			
**Psychometric measures**				

**GHQ**	**T0 [Mean ± SD]**	**T1 [Mean ± SD]**	**Mean change [95% CI]**	**Cohen's d**
Total Score	45.36 ± 8.53	49.08 ± 10.25	3.72 [1.72, 5.71]	0.42
Somatic Symptoms	12.05 ± 2.98	13.41 ± 2.89	1.36 [0.77, 1.96]	0.51
Anxiety/Insomnia	11.29 ± 3.80	13.06 ± 4.19	1.77 [0.81, 2.73]	0.42
Social Dysfunction	13.93 ± 2.56	14.40 ± 3.13	0.47 [-0.21, 1.16]	0.16
Severe Depression	7.91 ± 1.68	8.47 ± 2.44	0.57 [0.05, 1.08]	0.25
**PSS**				
Total Score	23.69 ± 5.46	25.26 ± 5.51	1.57 [0.50, 2.64]	0.33
**MIMIS Exposure**	56.32 ± 21.39	66.39 ± 19.70	10.08 [5.66, 14.49]	0.51
**PSQI item 4**	7.46 ± 0.91	6.77 ± 0.71	−0.69 [-0.89, −0.48]	−0.75

**Fig. 2:**
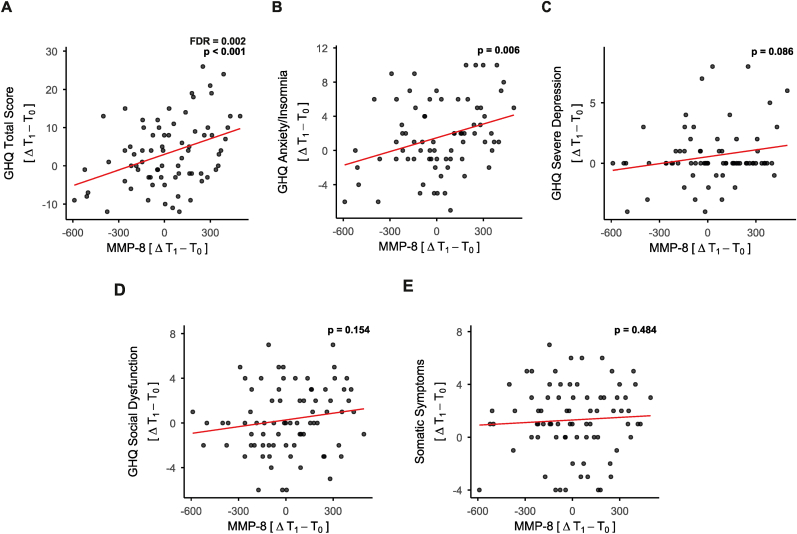
**Increased circulating matrix metalloproteinase-8 (MMP-8) levels are associated with general mental health symptoms and anxiety/insomnia.** Each data point represents an individual participant, with the x-axis showing the delta values for MMP-8 and the y-axis displaying the corresponding delta values for General Health Questionnaire (GHQ) total score and subscale scores. The red line represents the trend line derived from the linear regression analysis **A**: GHQ Total Score; **B**: GHQ Anxiety/Insomnia; **C**: GHQ Severe Depression; **D**: GHQ Social Dysfunction; **E**: GHQ Somatic Symptoms. Statistics: Linear regression analysis adjusting for age, BMI, gender, and smoking status. (For interpretation of the references to colour in this figure legend, the reader is referred to the Web version of this article.)

Based on prior evidence implicating inflammatory signaling in stress-related symptoms, we next conducted a hypothesis-driven regression analysis focusing on three predefined inflammatory markers: MMP-8, TNF-α and IL-6. Changes in circulating MMP-8 levels were significantly associated with changes in GHQ total scores over the three-month internship period (p < 0.001, FDR-corrected p = 0.002; Fig. 2A). Moreover, MMP-8 was significantly associated with changes in the GHQ Anxiety/Insomnia subscale (p = 0.006, Fig. 2B). To address potential conceptual overlap between sleep duration and the GHQ Anxiety/Insomnia subscale, we performed a sensitivity analysis including change in PSQI-4 scores as an additional covariate. The association between MMP-8 and Anxiety/Insomnia remained significant (p = 0.002), suggesting that the relationship was independent of changes in sleep duration. No significant associations were observed with the other GHQ subscales (Severe Depression [p = 0.086], Social Dysfunction [p = 0.154], or Somatic Symptoms [p = 0.484], Fig. 2C–E). To further account for within-subject dependence across time points, we additionally performed linear mixed-effects models including subject as a random effect (Supplementary Fig. 2). These analyses yielded comparable results: MMP-8 was significantly associated with GHQ total scores (p = 0.007) and with the Anxiety/Insomnia subscale (p = 0.044), supporting the robustness of the findings. Finally, we performed gender-stratified linear regression analyses. The association between changes in MMP-8 and GHQ total scores remained significant in females (N = 53, p = 0.001, FDR-corrected p = 0.003) but not in males (N = 22, p = 0.289, FDR-corrected p = 0.665; Supplementary Fig. 3), and the gender × ΔMMP-8 interaction was not statistically significant (p = 0.315).

Associations between GHQ scores and changes in TNF-α (Supplementary Fig. 4A–E) and IL-6 (Supplementary Fig. 5A–E) did not reach statistical significance. For TNF-α, the association with GHQ total score yielded p = 0.448 (FDR-corrected p = 0.672), with GHQ subscale p-values ≥0.294. For IL-6, the association with GHQ total score yielded p = 0.802 (FDR-corrected p = 0.802), with GHQ subscale p-values ≥ 0.099.

Next, we performed regression analyses to identify associations between GHQ total scores and the 97 circulating proteins in blood **(**Supplementary Table 1). Several inflammation-related proteins showed nominal associations (p < 0.05) with changes in GHQ scores (Supplementary Fig. 6A–G), including Lipopolysaccharide-binding protein (LBP) (p = 0.018, FDR-corrected p = 0.634), C-X-C motif chemokine ligand 10 (CXCL10) (p = 0.032, FDR-corrected p = 0.634), Prostaglandin-endoperoxide synthase 2 (PTGS2) (p = 0.039, FDR-corrected p = 0.634), and CD40 ligand (CD40L) (p = 0.040, FDR-corrected p = 0.634). Furthermore, A disintegrin and metalloproteinase with thrombospondin motifs 9 (ADAMTS9) (p = 0.033, FDR-corrected p = 0.634), Interleukin-4 (IL-4) (p = 0.039, FDR-corrected p = 0.634), and C-C motif chemokine ligand 11 (CCL11) (p = 0.046, FDR-corrected p = 0.634) were negatively associated with GHQ total score.

**Fig. 3:**
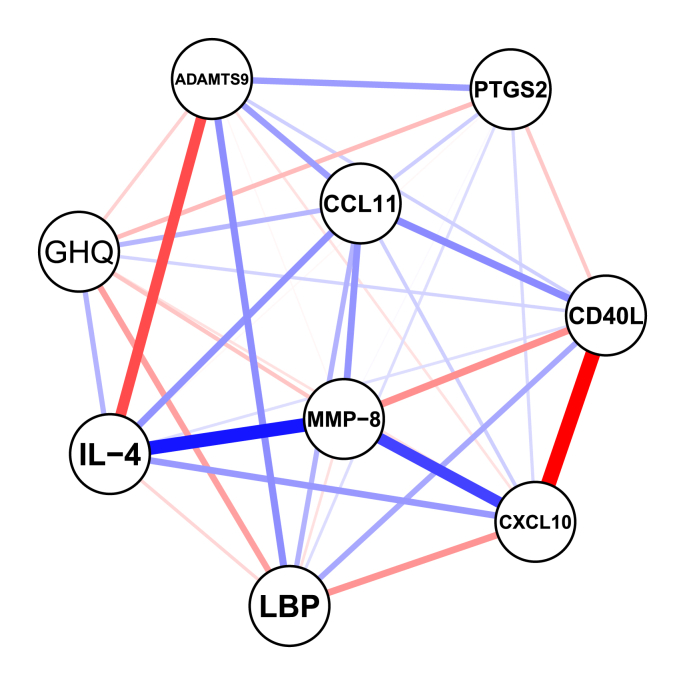
**Matrix metalloproteinase-8 (MMP-8) is a central node in the inflammatory network associated with general mental health symptoms.** The network includes proteins that showed nominally significant associations with general mental health symptoms assessed by the General Health Questionnaire (GHQ; red = positive connections; blue = negative connections). Abbreviations of the immune markers are listed in Supplementary Table 1. Statistics: Partial correlation analysis adjusting for age, BMI, gender, and smoking status. (For interpretation of the references to colour in this figure legend, the reader is referred to the Web version of this article.)

We visualized the network of immune markers associated with GHQ using network analysis (Fig. 3). Markers were included in the network if they showed a nominally significant association with GHQ (p < 0.05) in the linear regression analyses. In line with the regression results, MMP-8 emerged as a central node, directly linked to GHQ and connected to several other immune markers. In particular, it showed a positive association with CD40L (edge weight = 0.26), while negative associations were observed with IL-4 (edge weight = −0.55), CXCL10 (edge weight = −0.44), and CCL11 (edge weight = −0.25). In addition to the GHQ-MMP-8 connection, exploratory links were observed between GHQ and LBP, PTGS2, and ADAMTS9 (positive), as well as IL-4 and CCL11 (negative). These associations did not survive multiple-testing correction and should be considered hypothesis-generating. The case-dropping bootstrap revealed that both edge weights and node strength centrality were stable up to 28% of the sample being removed, with a correlation-stability coefficient of 0.28.

## 4 Discussion

Our study aimed to investigate how an ecological real-world stressor in the form of a medical internship was associated with general mental health in healthy medical students and how these symptoms are associated with circulating proteins of the immune system. In line with this aim, GHQ scores increased during the internship period, indicating a measurable rise in general mental health symptoms. This pattern is consistent with longitudinal findings in other occupational stress contexts. For example, Canal-Rivero et al. reported a significant worsening in GHQ somatic symptoms over a six-month follow-up in healthcare workers during the COVID-19 pandemic, with feelings of being overwhelmed at work emerging as a key predictor of psychological distress (Canal-Rivero et al., 2022). Similarly, higher occupational stress was associated with poorer general health as assessed by the GHQ in critical care nurses during the pandemic (Al-Ghabeesh et al., 2022). Moreover, studies using the GHQ in healthcare professionals have demonstrated elevated levels of psychological distress during periods of increased work-related burden (Comotti et al., 2023). In line with these observations, we found that perceived stress, as measured by the PSS, increased during the internship period. These findings are consistent with results from longitudinal and repeated-measures studies of healthy medical trainees facing academic and clinical demands. For instance, Dyrbye et al. reported significant increases in PSS scores over the course of a single academic year in first-year medical students, accompanied by declines in mental quality of life and happiness, and related these changes to the sustained pressures of medical training (Dyrbye et al., 2017). Repeated cross-sectional studies across four years of medical training have reported moderate to high levels of perceived stress, with increases during clinical rotations as students face shifting demands from academic workload to residency applications and career-related pressures (Musick et al., 2026). Studies of medical interns have likewise reported elevated perceived stress associated with occupational demands and personality traits (Chaillet et al., 2025). Together, these findings suggest that the observed increase in GHQ and PSS scores in our study population reflects a stress-related change in psychological well-being during the internship period.

In our hypothesis-driven analyses, we specifically tested whether MMP-8, TNF-α, and IL-6 were associated with increased GHQ scores. Of these markers, MMP-8 showed a significant positive association with GHQ total score. In addition, MMP-8 was significantly associated with the GHQ Anxiety/Insomnia subscale, suggesting a specific link between MMP-8 activity and stress-related sleep and anxiety symptoms. TNF-α and IL-6 were not significantly associated with GHQ scores.

In the exploratory analyses of the remaining 97 proteins, several markers showed nominal associations with GHQ scores. However, none survived FDR correction. Positive nominal associations were observed for LBP, CXCL10, PTGS2, and CD40L, whereas ADAMTS9, IL-4, and CCL11 showed nominal negative associations.

The exploratory network analysis broadly reflected the regression findings, with MMP-8 showing comparatively higher centrality and linking GHQ to several immune markers. However, these findings should be interpreted with caution. Given the modest sample size relative to the number of markers and the liberal regularization setting (tuning parameter = 0), the estimated network may include unstable or spurious edges. Consistent with this, the correlation stability coefficient (CS = 0.28) indicates limited robustness of centrality estimates. Therefore, the apparent prominence of MMP-8 should be regarded as descriptive and hypothesis-generating rather than mechanistic, and requires replication in larger samples.

MMP-8, also known as neutrophil collagenase, is a proteolytic enzyme mainly released by circulating myeloid cells such as neutrophils and monocytes (Birkedal-Hansen et al., 1993). Emerging evidence suggests that MMP-8 may be involved in stress-related inflammatory pathways and affective symptomatology. In a prospective longitudinal cohort of pregnant women, Meints et al. reported that circulating MMP-8 levels were positively associated with anxiety and depressive symptoms during early pregnancy and the postpartum period (Meints et al., 2014). This is consistent with our observation of a relationship between MMP-8 and anxiety/insomnia-related symptom dimensions, further supporting the concept that MMP-8 may reflect stress-associated inflammatory processes across different physiological states. In line with this, experimental studies further implicate MMP-8 in stress-related neurobiological processes. In a mouse model of chronic social defeat stress, increased MMP-8 contributed to alterations in the brain extracellular space, neurophysiological changes in the nucleus accumbens, and enhanced social avoidance behavior (Cathomas et al., 2024). Similarly, elevated MMP-8 levels were observed in the hippocampus and in peripheral blood of stress-exposed rats compared to non-exposed controls (Suzuki et al., 2023). Importantly, the translational relevance of these findings is supported by the same social defeat study, which demonstrated increased serum MMP-8 levels in patients with MDD compared to controls, with MMP-8 correlating with self-reported perceived stress (Cathomas et al., 2024).

While no study has yet investigated MMP-8 levels in healthy populations exposed to stress, several studies report increased levels of MMP-8 in patients with MDD and associations of MDD with a single-nucleotide polymorphism in the coding region of MMP-8 (Leday et al., 2018; Song et al., 2013). Compared to healthy controls, MDD was also associated with disinhibition of the immune pathway associated with MMP-8, even in the absence of elevated CRP (Sforzini et al., 2025). However, these findings stem predominantly from clinical samples and therefore do not clarify whether MMP-8 alterations are specific to manifest depressive disorder or may already be detectable at the level of stress-related symptom dimensions in non-clinical populations. Our findings extend this literature by suggesting that MMP-8 is associated not only with diagnosed affective disorders but also with anxiety/insomnia-related symptom burden in a healthy cohort, suggesting a dimensional rather than purely categorical relationship.

Interestingly, in exploratory gender-stratified analyses, the association between MMP-8 and GHQ total scores was significant in females but not males; however, the interaction test was non-significant. Although the present study was not specifically designed to test gender differences, this pattern could be of potential relevance given well-established gender differences in stress responsivity and immune regulation. Females generally exhibit stronger inflammatory responses to psychosocial stressors and show higher prevalence rates of anxiety and depressive symptoms (Martinez-Muniz and Wood, 2020; Bekhbat and Neigh, 2018), which may partly explain the stronger association observed in this subgroup. However, the number of male participants in our cohort was smaller, and future studies with larger and more balanced samples will be necessary to clarify whether the observed association truly differs by gender.

Given that MMP-8 can cleave tight junction proteins essential for maintaining blood-brain barrier (BBB) integrity (Parks et al., 2004; Van Lint and Libert, 2006) and that accumulating evidence links psychological stress to BBB disruption (Dion-Albert et al., 2022), stress-related increases in MMP-8 may represent a potential mechanistic bridge between peripheral inflammation and general mental health symptoms. Future studies should therefore investigate whether MMP-8-mediated alterations in BBB permeability contribute to anxiety- and sleep-related symptoms in both healthy and clinical populations.

Interestingly, the pro-inflammatory canonical cytokines TNF-α and IL-6 did not show significant associations with GHQ total score or subscales in our sample. Both markers are widely considered as central mediators of acute inflammatory stress responses and have repeatedly been reported to increase following laboratory-induced stress in healthy individuals and in stress-related psychiatric conditions. One possible explanation for the lack of correlation in our study is that TNF-α and IL-6 primarily reflect acute, transient inflammatory responses, whereas GHQ captures broader or more sustained symptomatology. Thus, these cytokines may be more sensitive to short-term or transient stress-related inflammatory responses, whereas GHQ may capture broader or more sustained symptom burden. It is also likely that other molecular pathways beyond classical systemic inflammation are more closely linked to variations in general mental symptoms in our sample. Further research is needed to clarify how different qualities, durations, and intensities of stress selectively influence specific inflammatory and non-inflammatory protein pathways.

Several limitations should be considered when interpreting our findings. First, while MMP-8 was examined as part of the hypothesis-driven analyses, the associations identified for the exploratory proteins reached significance only at a nominal (uncorrected) p < 0.05 and therefore carry an elevated risk of type I error. While such thresholds are acceptable for discovery proteomics and hypothesis generation, these findings should be viewed as exploratory and require confirmation in independent, larger cohorts of medical students, ideally with pre-specified targets, control of the false discovery rate, and orthogonal assay validation. Second, the observational design of the study does not allow for conclusions about causality. While we observed several associations between general mental health symptoms and immune markers, we cannot determine whether stress directly caused these changes. Although we conceptualized the internship as a period of increased psychosocial demands, stress was not experimentally manipulated but assessed using self-report measures (PSS, MIMIS). Therefore, the internship itself should not be interpreted as a uniform stressor for all participants. Future studies would need to include a control group that does not experience any stress in order to draw further conclusions. Third, general mental health symptoms were assessed using a self-report questionnaire, which may be influenced by subjective interpretation or reporting bias. In addition, participants’ physical and psychiatric health status was based on self-report and study eligibility criteria rather than a formal clinical evaluation by a physician, and therefore undiagnosed conditions cannot be fully excluded. Fourth, the analysis was limited to two time points, which may not fully reflect the dynamic nature of stress and immune function over a longer period. Fifth, although changes in PSS scores, MIMIS exposure, and sleep duration between baseline and follow-up indicate increased stress in participants, the internship may not have been an adequate stressor for all students and observed increases in PSS values may as well relate to other (not assessed) real-life stressors. In addition, seasonal influences or other time-related effects cannot be excluded as alternative explanations for the observed T0-T1 changes. Thus, future studies could aim to focus on periods of high-intensity, real-life stress (e.g. start of residency in medical graduates). Additionally, the overrepresentation of female participants, a result of the high number of female medical students, limits the generalizability of our findings. Also important with respect to generalizability is that the cohort of medical students represents a rather educated population. Furthermore, willingness to participate in the study could also lead to a selection bias within the student population. Lastly, although we controlled for several confounding variables, including age, gender, BMI, and smoking status, other factors such as nutrition, physical activity, or underlying health conditions were not systematically assessed and could have influenced the results.

The present findings may have clinical relevance for understanding how psychological stress contributes to health problems in students and other populations exposed to work-related stress. The reported blood signature suggests a biological shift that could increase vulnerability to stress-related conditions, both mental and somatic. While the observed changes were modest, they reflect early immune alterations that may foreshadow more overt symptoms. Monitoring stress-related immune markers in high-risk groups, such as medical students, could help identify individuals who may benefit from early interventions, such as stress reduction programs, lifestyle adjustments, or targeted support. Our data also support the idea that mental health and immune function are closely linked, and that psychological well-being should be considered an important factor in preventive health strategies. However, whether stress-reduction or occupational interventions can meaningfully alter immune markers or psychological outcomes should be examined in future studies that include appropriate control groups and, ideally, randomized intervention designs.

## CRediT authorship contribution statement

**Jasmin Ewert:** Writing – review & editing, Writing – original draft, Visualization, Investigation, Formal analysis. **Laura Meine:** Writing – review & editing, Investigation. **Ella McPherson:** Writing – review & editing, Investigation. **Sarah Fehr:** Writing – review & editing, Investigation. **Tobias R. Spiller:** Writing – review & editing, Visualization, Formal analysis. **Benedikt Dechow:** Writing – review & editing, Investigation. **Gaia Ferrante:** Writing – review & editing, Investigation. **Sebastian Hachenberg:** Writing – review & editing, Investigation. **Lidya Aslan:** Writing – review & editing, Investigation. **Antonia Lüönd:** Formal analysis, Writing – review & editing. **Bogdan Rivoal Mateescu:** Writing – review & editing, Investigation. **Birgit Kleim:** Writing – review & editing, Funding acquisition, Conceptualization. **Flurin Cathomas:** Writing – review & editing, Writing – original draft, Supervision, Funding acquisition, Formal analysis, Conceptualization.

## Ethics approval and consent to participate

Ethics approval for the study was obtained from the Cantonal Ethics Commission of Zurich (KEK, 2022-01169) and informed consent was obtained from all study participants.

## Declaration of generative AI and AI-assisted technologies in the manuscript preparation process

During the preparation of this work the authors used ChatGPT/OpenAI in order to spell-check the manuscript. After using this tool, the authors reviewed and edited the content as needed and take full responsibility for the content of the published article.

## Declaration of competing interest

The authors declare the following financial interests/personal relationships which may be considered as potential competing interests: Flurin Cathomas reports financial support was provided by Swiss Life Jubiläumsstiftung für Volksgesundheit und medizinische Forschung. Birgit Kleim reports financial support was provided by University of Zurich. If there are other authors, they declare that they have no known competing financial interests or personal relationships that could have appeared to influence the work reported in this paper.

## Data Availability

Data will be made available on request.
