# Supplementary figures and images for: Psychological stress during medical internship is associated with inflammatory signatures linked to mental health
Source: Brain Behav Immun Health. 2026 Apr 18;54:101243. doi: 10.1016/j.bbih.2026.101243 (PMC13141554; doi:10.1016/j.bbih.2026.101243)

**A**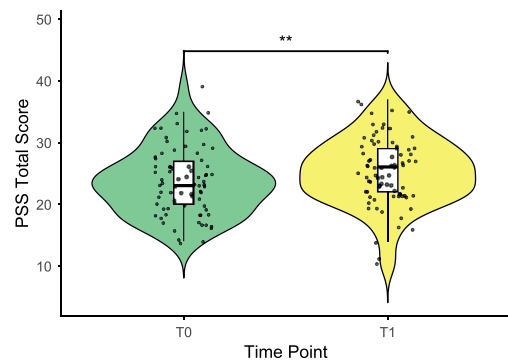**B**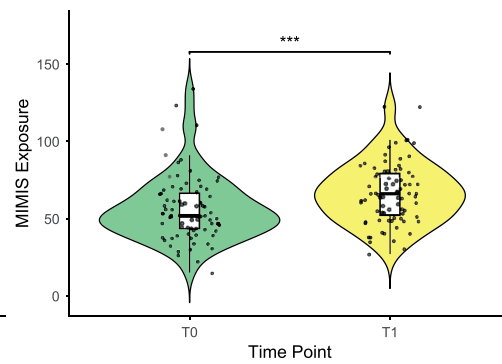**C**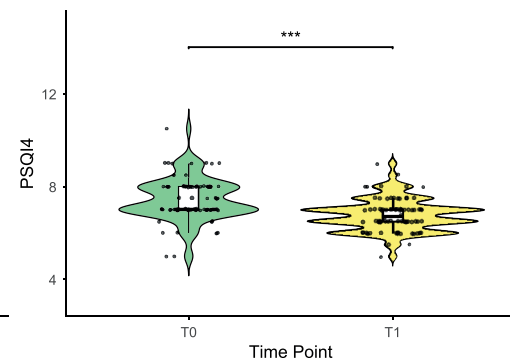

Supplement: Multimedia component 1 [file mmc1.pdf]

**A**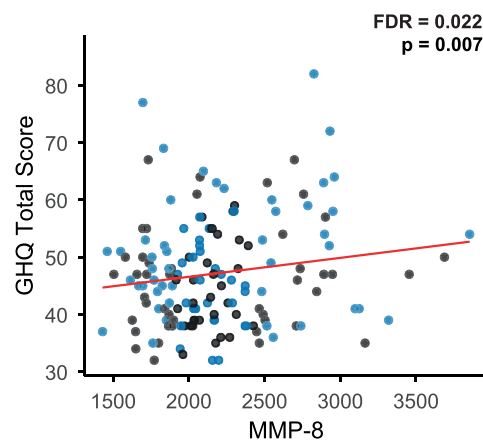**B**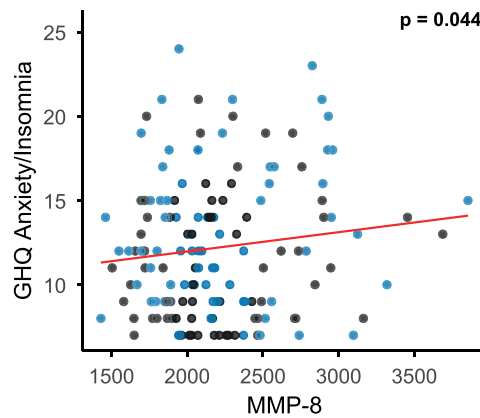**C**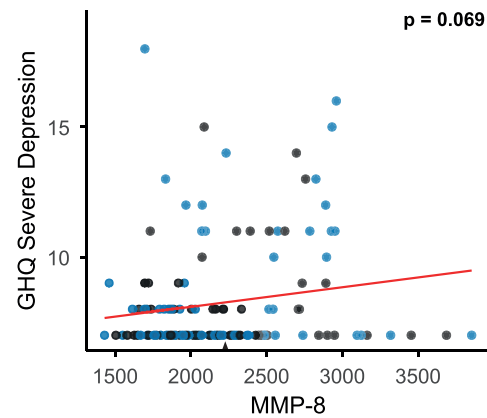**D**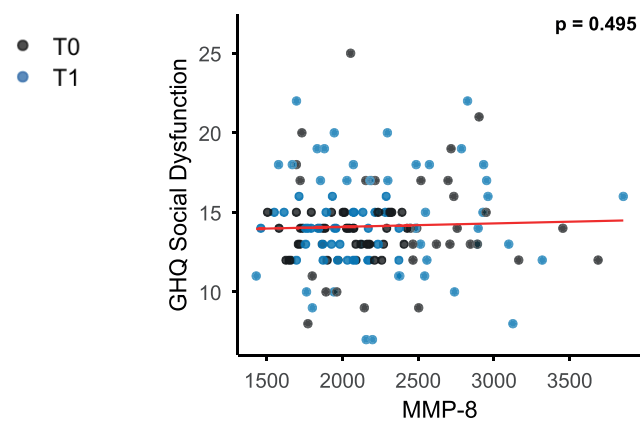**E**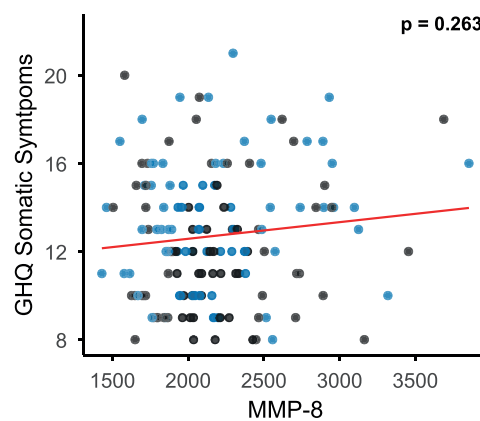

Supplement: Multimedia component 2 [file mmc2.pdf]

A

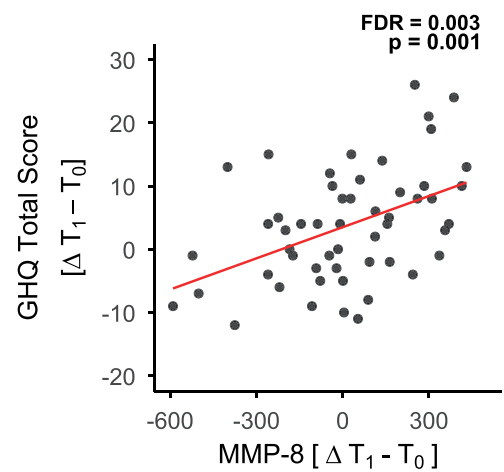

B

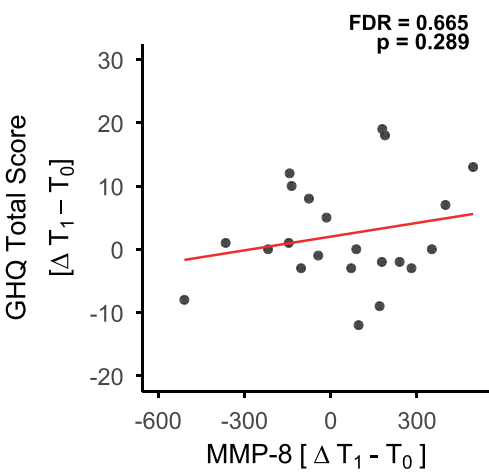

Supplement: Multimedia component 3 [file mmc3.pdf]

**A**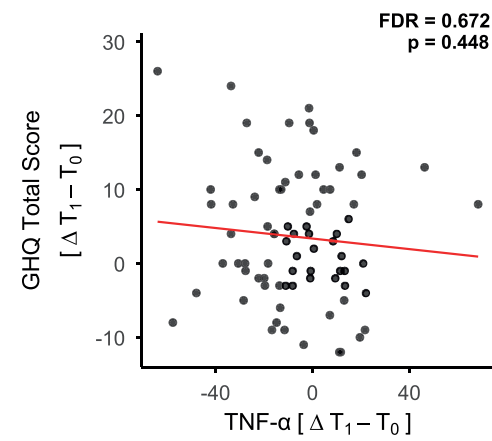**B**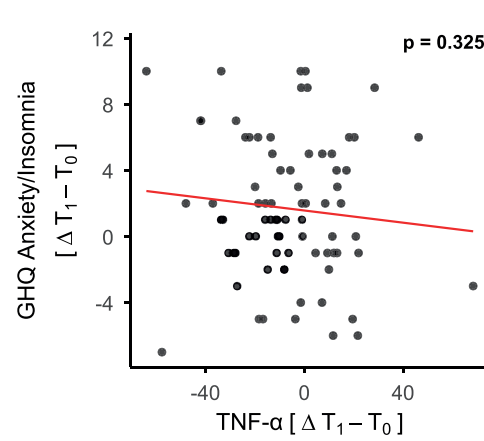**C**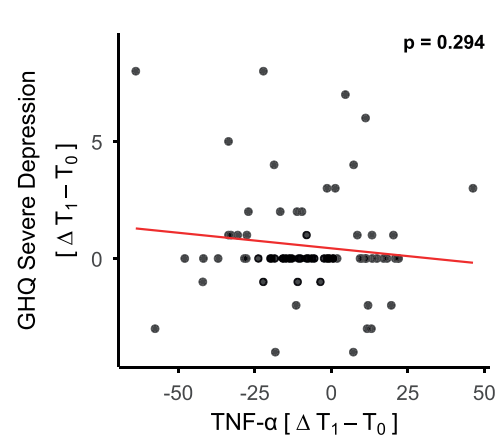**D**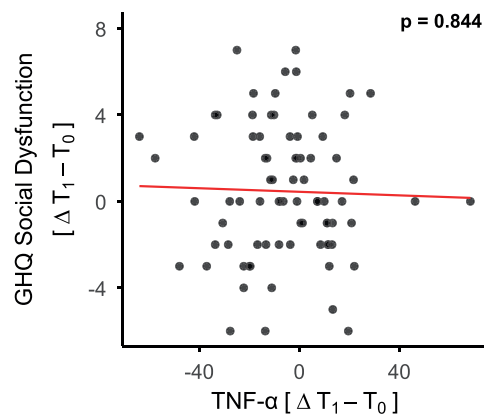**E**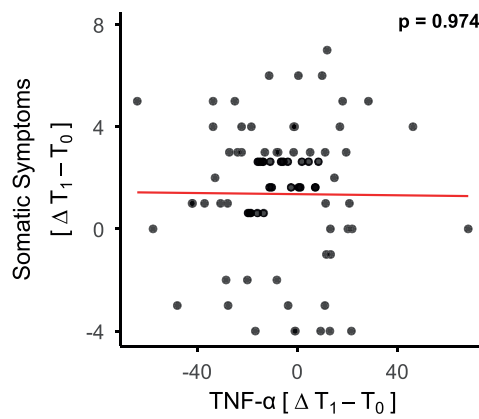

Supplement: Multimedia component 4 [file mmc4.pdf]

**A**

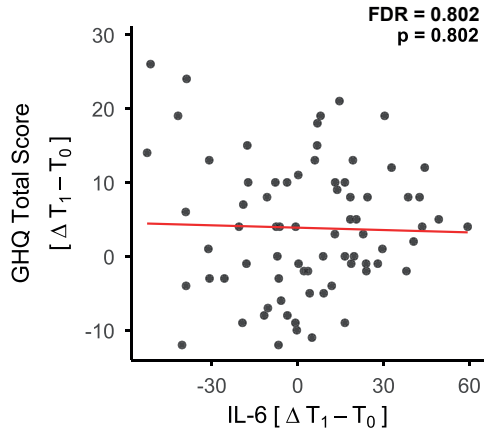

**B**

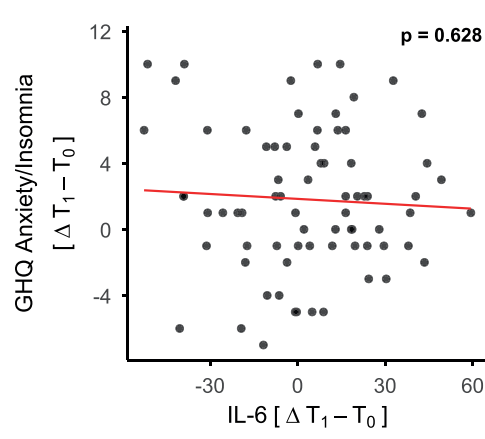

**C**

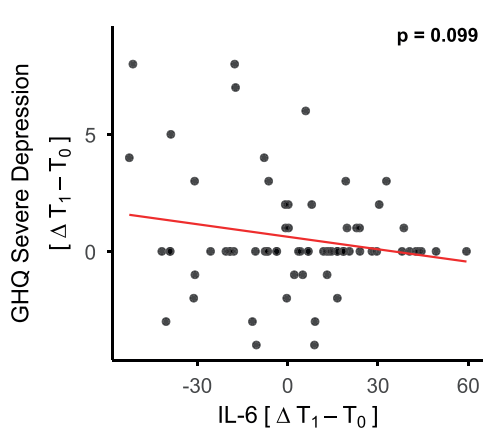

**D**

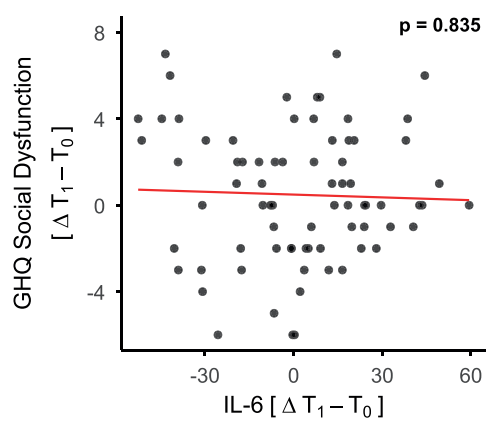

**E**

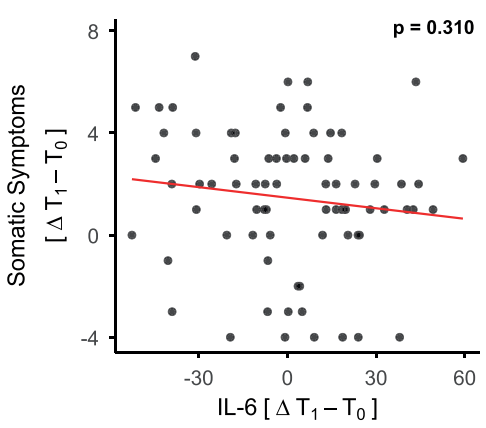

Supplement: Multimedia component 5 [file mmc5.pdf]

**A**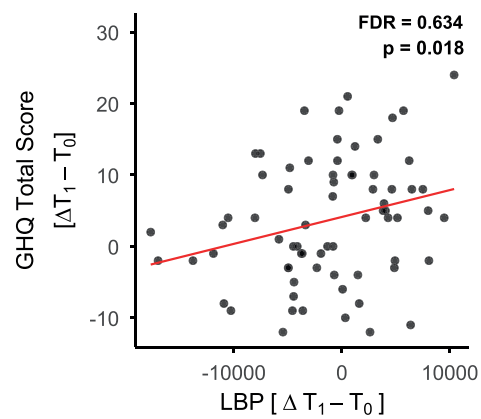**B**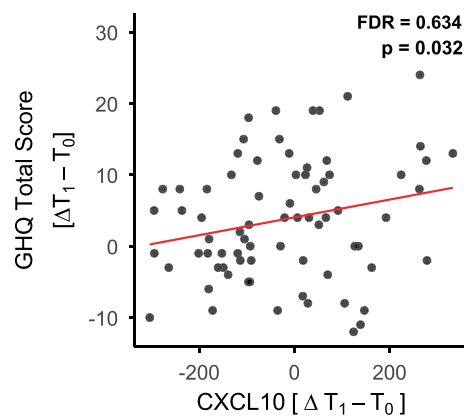**C**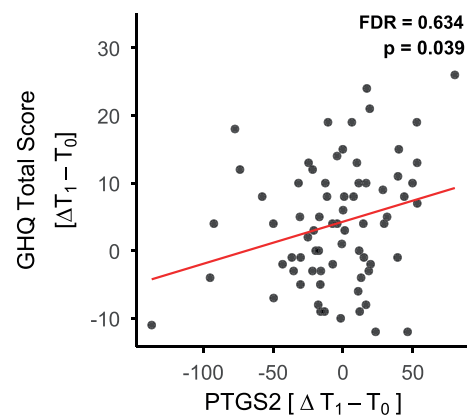**D**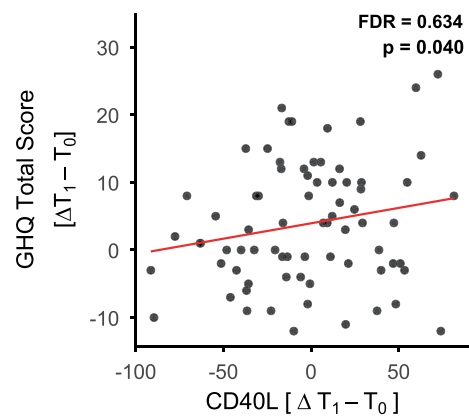**E**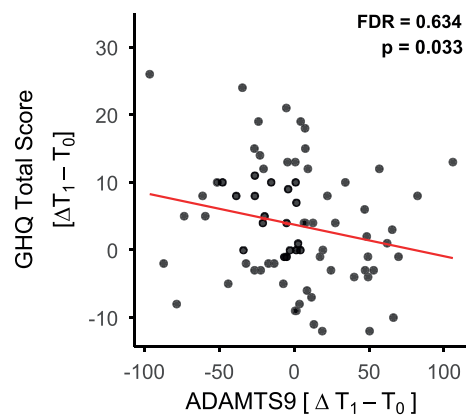**F**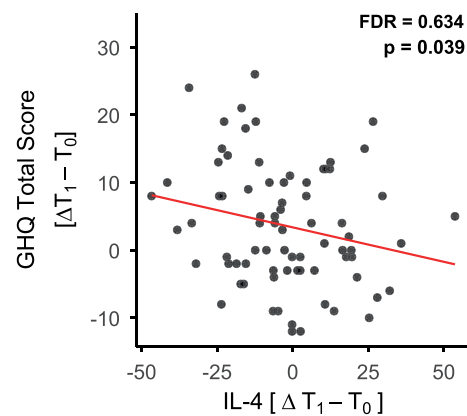**G**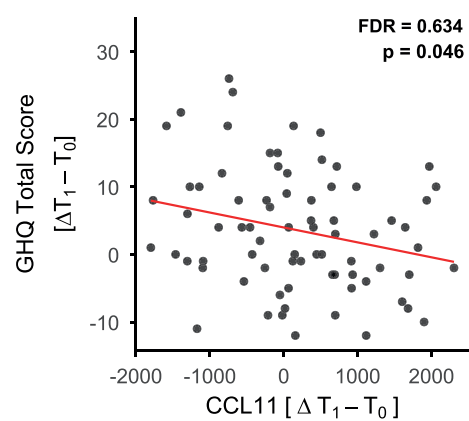

Supplement: Multimedia component 6 [file mmc6.pdf]
