# Supplementary material for: Psychological stress during medical internship is associated with inflammatory signatures linked to mental health
Source: Brain Behav Immun Health. 2026 Apr 18;54:101243. doi: 10.1016/j.bbih.2026.101243 (PMC13141554; doi:10.1016/j.bbih.2026.101243)
Supplement: Multimedia component 7 [file mmc7.docx]

| Analyte | Abbreviation | N | β | p-value | FDR-corrected p-value |
| --- | --- | --- | --- | --- | --- |
| *Pro-inflammatory cytokines* | | | | | |
| Interleukin-1 alpha | IL1α | 74 | -0.034 | 0.777 | 0.928 |
| Interleukin-1 beta | IL-1β | 76 | 0.077 | 0.511 | 0.840 |
| Interleukin-6 | IL-6 | 74 | -0.030 | 0.802 | 0.802^a^ |
| Interleukin-12 | IL-12 | 75 | 0.159 | 0.181 | 0.771 |
| Interleukin-17A | IL-17A | 76 | 0.012 | 0.919 | 0.960 |
| Interleukin-18 | IL-18 | 76 | 0.124 | 0.286 | 0.771 |
| Tumor necrosis factor | TNF-α | 77 | -0.088 | 0.448 | 0.672^a^ |
| Oncostatin-M | OSM | 75 | 0.066 | 0.584 | 0.851 |
| *Anti-inflammatory / regulatory cytokines* | | | | | |
| Interleukin-4 | IL-4 | 76 | -0.235 | 0.039 | 0.634 |
| Interleukin-10 | IL-10 | 73 | -0.010 | 0.933 | 0.963 |
| Transforming growth factor beta-1 | TGF-β | 75 | 0.191 | 0.108 | 0.755 |
| Transforming growth factor beta-2 | TGF-β2 | 74 | 0.152 | 0.189 | 0.771 |
| Transforming growth factor beta-3 | TGF-β3 | 77 | 0.127 | 0.277 | 0.771 |
| Annexin A1 | ANXA1 | 75 | 0.159 | 0.178 | 0.771 |
| *Interferons* | | | | | |
| Interferon alpha-1/13 | IFN-α | 77 | -0.007 | 0.953 | 0.969 |
| Interferon beta | IFN-β | 76 | 0.149 | 0.218 | 0.771 |
| Interferon gamma | IFN-γ | 75 | -0.213 | 0.067 | 0.662 |
| *CC chemokines* | | | | | |
| C-C motif chemokine 2 | CCL2 | 75 | 0.114 | 0.330 | 0.774 |
| C-C motif chemokine 3 | CCL3 | 76 | -0.139 | 0.252 | 0.771 |
| C-C motif chemokine 4 | CCL4 | 75 | 0.065 | 0.588 | 0.851 |
| C-C motif chemokine 5 | CCL5 | 73 | 0.066 | 0.584 | 0.851 |
| C-C motif chemokine 7 | CCL7 | 76 | -0.006 | 0.958 | 0.969 |
| Eotaxin | CCL11 | 75 | -0.234 | 0.046 | 0.634 |
| *CXC chemokines* | | | | | |
| Growth-regulated alpha protein | CXCL1 | 74 | 0.128 | 0.286 | 0.771 |
| Gro-beta | CXCL2 | 73 | 0.189 | 0.109 | 0.755 |
| Gro-gamma | CXCL3 | 72 | 0.148 | 0.223 | 0.771 |
| Platelet factor 4 | CXCL4 | 71 | 0.077 | 0.533 | 0.851 |
| C-X-C motif chemokine 5 | CXCL5 | 76 | -0.034 | 0.776 | 0.928 |
| C-X-C motif chemokine 6 | CXCL6 | 74 | 0.119 | 0.336 | 0.774 |
| Neutrophil-activating peptide 2 | CXCL7 | 72 | 0.110 | 0.367 | 0.774 |
| C-X-C motif chemokine 10 | CXCL10 | 74 | 0.249 | 0.032 | 0.634 |
| Interleukin-8 | IL-8 | 77 | -0.108 | 0.350 | 0.774 |
| *Growth factors* | | | | | |
| Vascular endothelial growth factor A | VEGF-A | 73 | 0.134 | 0.263 | 0.771 |
| Fibroblast growth factor 2 | FGF2 | 73 | 0.039 | 0.742 | 0.928 |
| Fibroblast growth factor 21 | FGF21 | 76 | -0.023 | 0.848 | 0.928 |
| Fibroblast growth factor 22 | FGF22 | 75 | 0.028 | 0.809 | 0.928 |
| beta-nerve growth factor | β-NGF | 75 | 0.044 | 0.707 | 0.928 |
| Brain-derived neurotrophic factor | BDNF | 73 | 0.106 | 0.372 | 0.774 |
| Glial cell line-derived neurotrophic factor | GDNF | 72 | 0.041 | 0.743 | 0.928 |
| Granulocyte colony-stimulating factor | G-CSF | 75 | 0.096 | 0.408 | 0.785 |
| Thrombopoietin | THPO | 75 | 0.068 | 0.562 | 0.851 |
| Insulin-like growth factor I | IGF-I | 74 | 0.110 | 0.363 | 0.774 |
| *Matrix remodeling enzymes* | | | | | |
| 72 kDa type IV collagenase | MMP-2 | 74 | 0.143 | 0.228 | 0.771 |
| Stromelysin-1 | MMP-3 | 74 | 0.041 | 0.731 | 0.928 |
| Matrilysin | MMP-7 | 74 | 0.023 | 0.848 | 0.928 |
| Neutrophil collagenase | MMP-8 | 75 | 0.383 | <0.001 | 0.002^a^ |
| Matrix metalloproteinase-9 | MMP-9 | 74 | -0.050 | 0.677 | 0.928 |
| Macrophage metalloelastase | MMP-12 | 74 | -0.034 | 0.786 | 0.928 |
| Collagenase 3 | MMP-13 | 75 | 0.103 | 0.384 | 0.774 |
| A disintegrin and metalloproteinase with thrombospondin motifs 1 | ADAMTS1 | 75 | 0.148 | 0.208 | 0.771 |
| A disintegrin and metalloproteinase with thrombospondin motifs 4 | ADAMTS4 | 75 | 0.021 | 0.861 | 0.928 |
| A disintegrin and metalloproteinase with thrombospondin motifs 5 | ADAMTS5 | 74 | 0.223 | 0.064 | 0.662 |
| A disintegrin and metalloproteinase with thrombospondin motifs 9 | ADAMTS9 | 75 | -0.252 | 0.033 | 0.634 |
| A disintegrin and metalloproteinase with thrombospondin motifs 12 | ADAMTS12 | 75 | 0.013 | 0.913 | 0.960 |
| A disintegrin and metalloproteinase with thrombospondin motifs 13 | ADAMTS13 | 75 | 0.081 | 0.486 | 0.832 |
| Metalloproteinase inhibitor 1 | TIMP-1 | 77 | 0.211 | 0.068 | 0.662 |
| Metalloproteinase inhibitor 2 | TIMP-2 | 73 | 0.072 | 0.542 | 0.851 |
| Metalloproteinase inhibitor 3 | TIMP-3 | 74 | 0.106 | 0.380 | 0.774 |
| Metalloproteinase inhibitor 4 | TIMP-4 | 75 | -0.037 | 0.765 | 0.928 |
| *Adhesion molecules* | | | | | |
| Annexin A2 | ANXA2 | 72 | 0.081 | 0.497 | 0.832 |
| Vascular cell adhesion protein 1 | VCAM-1 | 76 | 0.026 | 0.826 | 0.928 |
| Platelet endothelial cell adhesion molecule | PECAM-1 | 74 | -0.094 | 0.425 | 0.785 |
| E-selectin | E-Selectin | 73 | -0.032 | 0.790 | 0.928 |
| P-selectin | P-Selectin | 71 | 0.058 | 0.635 | 0.892 |
| CD40 ligand | CD40L | 74 | 0.240 | 0.040 | 0.634 |
| von Willebrand factor | vWF | 73 | 0.199 | 0.096 | 0.756 |
| Fibronectin | FN1 | 74 | 0.076 | 0.546 | 0.851 |
| Thrombospondin-2 | THBS2 | 75 | 0.176 | 0.130 | 0.771 |
| *Complement system* | | | | | |
| Complement C3 | C3 | 73 | 0.022 | 0.859 | 0.928 |
| Complement C4 | C4 | 73 | 0.022 | 0.858 | 0.928 |
| C5a anaphylatoxin | C5a | 74 | 0.133 | 0.268 | 0.771 |
| *Acute phase and innate immune proteins* | | | | | |
| C-reactive protein | CRP | 72 | 0.085 | 0.493 | 0.832 |
| Serum amyloid A-1 protein | SAA1 | 73 | -0.132 | 0.269 | 0.771 |
| Serum amyloid A-2 protein | SAA2 | 74 | -0.096 | 0.425 | 0.785 |
| Ferritin | FTH1 | 76 | 0.002 | 0.987 | 0.987 |
| Lipopolysaccharide-binding protein | LBP | 71 | 0.278 | 0.018 | 0.634 |
| High mobility group protein B1 | HMGB1 | 72 | -0.110 | 0.355 | 0.774 |
| High mobility group protein B2 | HMGB2 | 72 | 0.111 | 0.357 | 0.774 |
| Protein S100-A11 | S100A11 | 73 | 0.086 | 0.466 | 0.821 |
| Protein S100-A4 | S100A4 | 72 | 0.137 | 0.259 | 0.771 |
| *Neutrophil / myeloid enzymes and oxidative pathways* | | | | | |
| Neutrophil elastase | ELA2 | 75 | -0.117 | 0.338 | 0.774 |
| Myeloperoxidase | MPO | 73 | -0.071 | 0.565 | 0.851 |
| iNOS | iNOS | 74 | -0.169 | 0.145 | 0.771 |
| NOS | NOS | 74 | 0.134 | 0.268 | 0.771 |
| COX-1 | COX-1 | 72 | 0.107 | 0.391 | 0.774 |
| Prostaglandin G/H synthase 2 | PTGS2 | 71 | 0.247 | 0.039 | 0.634 |
| *Neuro-axonal and glial injury markers* | | | | | |
| Neurofilament light polypeptide | NfL | 74 | 0.029 | 0.808 | 0.928 |
| Glial fibrillary acidic protein | GFAP | 76 | 0.012 | 0.920 | 0.960 |
| *Neuroendocrine and metabolic hormones* | | | | | |
| Insulin | INS | 76 | 0.156 | 0.176 | 0.771 |
| Leptin | LEP | 74 | 0.091 | 0.437 | 0.785 |
| Appetite-regulating hormone | Ghrelin | 74 | 0.102 | 0.389 | 0.774 |
| Lipoprotein lipase | LPL | 73 | 0.061 | 0.607 | 0.866 |
| Apolipoprotein(a) | Apo(a) | 74 | -0.029 | 0.805 | 0.928 |
| Corticotropin | ACTH | 75 | 0.139 | 0.237 | 0.771 |
| Thyroid stimulating hormone | TSH | 74 | -0.095 | 0.433 | 0.785 |
| FK506-binding protein 5 | FKBP5 | 72 | 0.130 | 0.281 | 0.771 |
| *Tryptophan-Kynurenine pathway* | | | | | |
| Indoleamine 2,3-dioxygenase 1 | IDO-1 | 75 | -0.194 | 0.096 | 0.755 |
| Tryptophan 2,3-dioxygenase | TDO | 77 | -0.162 | 0.155 | 0.771 |
| *Inflammasome* | | | | | |
| NACHT, LRR and PYD domains-containing protein 3 | NLRP3 | 74 | 0.038 | 0.753 | 0.928 |
| *Other signaling molecules* | | | | | |
| Neuropeptide Y | NPY | 74 | 0.040 | 0.733 | 0.928 |

ᵃFalse discovery rate correction (Benjamini-Hochberg) was performed only across the three predefined hypothesis-driven markers (MMP-8, TNF-α and IL-6).

**Supplementary Table 1.** List of 100 proteins analyzed using the SomaLogic panel, including the number of participants included in the linear regression analyses for each analyte and the General Health Questionnaire (GHQ). For each analyte, standardized regression coefficients (β), uncorrected p-values, and false discovery rate (FDR)-corrected p-values are reported. Outliers exceeding ±2 standard deviations were excluded from the analyses.
