# Supplementary material for: Psychological stress during medical internship is associated with inflammatory signatures linked to mental health
Source: Brain Behav Immun Health. 2026 Apr 18;54:101243. doi: 10.1016/j.bbih.2026.101243 (PMC13141554; doi:10.1016/j.bbih.2026.101243)
Supplement: Multimedia component 8 [file mmc8.docx]

**Supplementary Figure 1. Increased perceived stress and microstressor exposure, and reduced sleep duration after 3 months of a medical internship.** The violin plots illustrate the density of individual data points, with an overlaid boxplot indicating the median, interquartile range, and overall spread of the data at baseline (T0) and after 3 months of the internship (T1). **A**: Perceived Stress Scale (PSS); **B:** Mainz Inventory of Microstressors (MIMIS) Exposure; **C**: Pittsburgh Sleep Quality Index (PSQI) item 4. Statistics: Paired two-sided t-test (**p < 0.01, ***p < 0.001).

**Supplementary Figure 2. Increased circulating matrix metalloproteinase-8 (MMP-8) levels are associated with increased general mental health symptoms.** Each data point represents one observation from an individual participant at baseline (T0) or follow-up (T1), with the x-axis showing circulating MMP-8 levels and the y-axis displaying the corresponding total and subscales of the General Health Questionnaire (GHQ). Black data points represent T0, and blue data points represent T1. The red line represents the fitted regression line. **A**: GHQ Total Score; **B**: GHQ Anxiety/Insomnia; **C**: GHQ Severe Depression; **D**: GHQ Social Dysfunction; **E**: GHQ Somatic Symptoms. Statistics: Linear mixed-effects models including time point (T0/T1) as a repeated factor and adjusting for age, BMI, gender, and smoking status, with random intercepts for participants.

**Supplementary Figure 3. Association between matrix metalloproteinase-8 (MMP-8) and** **general mental health symptoms stratified by gender.** Each data point represents an individual participant, with the x-axis showing the delta value for MMP-8 and the y-axis displaying the corresponding delta value for the General Health Questionnaire (GHQ). The red line represents the trend line derived from the linear regression analysis. **A**: Females; **B**: Males. Statistics: Gender-stratified linear regression analyses adjusting for age, BMI, and smoking status.

**Supplementary Figure 4*.* Tumor necrosis factor-α (TNF-α) is not associated with general mental health symptoms.** Each data point represents an individual participant, with the x-axis showing the delta value for TNF-α and the y-axis displaying the corresponding delta values for total and subscale of the General Health Questionnaire (GHQ). The red line represents the trend line derived from the linear regression analysis. **A**: GHQ Total Score; **B**: GHQ Anxiety/Insomnia; **C**: GHQ Severe Depression; **D**: GHQ Social Dysfunction; **E**: GHQ Somatic Symptoms. Statistics: Linear regression analysis adjusting for age, BMI, gender, and smoking status.

**Supplementary Figure 5*.* Interleukin-6 (IL-6) is not associated with general mental health symptoms.** Each data point represents an individual participant, with the x-axis showing the delta value for IL-6 and the y-axis displaying the corresponding delta values for total and subscales of the General Health Questionnaire (GHQ). The red line represents the trend line derived from the linear regression analysis. **A**: GHQ Total Score; **B**: GHQ Anxiety/Insomnia; **C**: GHQ Severe Depression; **D**: GHQ Social Dysfunction; **E**: GHQ Somatic Symptoms. Statistics: Linear regression analysis adjusting for age, BMI, gender, and smoking status.

**Supplementary Figure 6. Individual proteins associated with changes in general mental health.** Each data point represents an individual participant, with the x-axis showing the delta value for the circulating protein and the y-axis displaying the corresponding delta value for total scores of the General Health Questionnaire (GHQ). The red line represents the trend line derived from the linear regression analysis. Abbreviations of the immune markers are listed in **Supplementary Table 1**. **A**: LBP; **B**: CXCL10; **C**: PTGS2; **D**: CD40L; **E**: ADAMTS9; **F**: IL-4; **G**: CCL11. Statistics: Linear regression analysis adjusting for age, BMI, gender, and smoking status.

**Table 1. Demographic and health-related characteristics of the study sample (N = 82).** The table summarizes demographic variables including age, body mass index (BMI), gender distribution, and smoking status, as well as psychometric measures assessed at baseline (T0) and follow-up (T1), including the General Health Questionnaire (GHQ) and GHQ subscales, the Perceived Stress Scale (PSS), Mainz Inventory of Microstressors (MIMIS) Exposure, and Pittsburgh Sleep Quality Index (PSQI)-4. Values are presented as mean ± standard deviation (SD). Cohen’s d for paired comparisons (T1–T0) is reported as a measure of effect size.
